# Revisiting an open access monograph experiment: measuring citations and tweets 5 years later

**DOI:** 10.1007/s11192-016-2160-6

**Published:** 2016-10-17

**Authors:** Ronald Snijder

**Affiliations:** 1 OAPEN Foundation, The Hague, The Netherlands; 2 Universiteit Leiden, Leiden, The Netherlands

**Keywords:** Open access, Monographs, Citations, Altmetrics, Tweets

## Abstract

An experiment run in 2009 could not assess whether making monographs available in open access enhanced scholarly impact. This paper revisits the experiment, drawing on additional citation data and tweets. It attempts to answer the following research question: does open access have a positive influence on the number of citations and tweets a monograph receives, taking into account the influence of scholarly field and language? The correlation between monograph citations and tweets is also investigated. The number of citations and tweets measured in 2014 reveal a slight open access advantage, but the influence of language or subject should also be taken into account. However, Twitter usage and citation behaviour hardly overlap.

## Introduction

While the question whether publishing in open access (OA) leads to a citation advantage has been studied numerous times for journal articles, much less work has been done in the realm of monographs. This imbalance is further illustrated by the fact that literature on articles is listed in several overviews—for instance by Archambault et al. (2014, 2016) or SPARC Europe (2015)—while publications on monographs are scarce.

The impact of scholarly publications has traditionally been assessed through citations, and, more recently, altmetrics have come into use as another type of impact measure. Here, altmetrics are defined as the measurement of online activities about scholarly publications. A specific form of altmetrics—Twitter mentions—will be used as an indicator of societal rather than academic impact of scholarly books.

Until recently, books have been largely ignored by those attempting to measure impact: both in the realm of citations and altmetrics. This paper will address this lacuna by analysing the role of open access on the impact of books, based on experimental data.

In 2009, an experiment was conducted on 400 monographs, measuring the effects of open access on discovery, online consultation, sales figures, dissemination channels and citations (Snijder 2010). In line with expectations, the experiment found that making books freely available enhances discoverability and online consultation. Furthermore, no significant influence on sales could be established. These outcomes are consistent with the results of other investigations (Ferwerda et al. 2013; Snijder 2014).

The experiment was less successful in establishing whether OA enhances the scholarly impact of books in a more traditional sense: through citations. Revisiting the experiment will help to answer this question. At the conclusion of the 2009 experiment no citation advantage for freely accessible books could be found. This is in contrast to journal articles, where higher citation rates for OA have been frequently reported. In October 2014 citations of the 400 monographs included in the original experiment were measured again, this time combined with the number of tweets mentioning each book.

In 2009 it was not possible to assess whether making monographs freely available enhanced scholarly impact, nor could anything be said about influence on society at large. This paper revisits the experiment, drawing on additional citation data as well as developments in the altmetrics landscape. It attempts to answer the following research question: does open access have a positive influence on the number of citations and tweets a monograph receives, taking into account the influence of scholarly field and language? Furthermore, looking into the correlation between monograph citations and tweets helps to determine whether these measurements are related.

## Background

This review focuses on monographs, starting with monograph citations before discussing alternative impact metrics as they relate to books. Apart from availability, two other factors may influence citations and altmetrics uptake: scholarly field and language. Different citation cultures exist within individual fields of study, making it hard to compare bibliometrics data between disciplines without normalisation.^1^ Furthermore, some authors suspect a bias towards English language publications in citation databases; this will be discussed in “The influence of language” section. The language of the publications included in the experiment discussed in this paper may affect its outcomes, as roughly half of the books included in the study—178 books—are written in English; the remaining 212 books were written in Dutch or other languages.

Another recurring theme in the literature on OA is the correlation between citations and altmetrics, see for instance Thelwall et al. (2013). If altmetrics are closely connected to scholarly impact, one might expect a statistically significant correlation between them. On the other hand, when altmetrics are seen as measuring a different type of interest in scholarly output—rather than as a proxy for citations—it may be more useful to search for online activity relating to scholarly books with the weakest correlation to citations. In that way, the broadest possible spectrum of engagement with monographs may be captured. This is discussed further in “What is the relation between citations and altmetrics?” section.

One of the assumptions of the original 2009 experiment was that making monographs available in open access enables more researchers to read books that would otherwise be inaccessible. The results of the experiment pointed to significantly greater usage—discoverability and online consultation—for freely available books. It was assumed that enhanced access would also lead to more citations, as it made books available to scholars working in more restrained environments. This assumption is challenged by the findings of a survey of 2231 humanities and social science researchers based in the United Kingdom: only ten percent of the respondents reported having difficulties in accessing monographs (OAPEN-UK 2014).

This perception by researchers opens interesting possibilities. If professional users of monographs have no serious problems in accessing them, we would expect to find a smaller citation advantage for OA books, or none at all. However, among the outcomes of the 2010 experiment was the improved discovery and online consultation of free online books. We might assume that a significant part of that online usage is coming from readers other than academics. In the discussion of altmetrics outlets, tweets are strongly associated with the wider public (Bornmann 2014; Haustein et al. 2014). For readers not connected to universities with large library collections, open access has direct benefits, potentially leading to more mentions on Twitter and the wider dissemination of research.

The OAPEN-UK project also looked into researchers’ attitudes towards making their books freely accessible. It concluded that authors see open access publishing as a way to increase their readership, and that this perceived benefit of open access is valued by many. However, opinions differ about the way it should be implemented (Collins and Milloy 2016).

### Citations and books

Glänzel and Schoepflin (1999) discussed the differences in citation behaviour in the humanities and social sciences compared to the sciences. They matched the percentage of cited articles to citations to books and other long form publication. In scientific fields such as immunology or solid-state physics, the amount of cited articles is over 85 %. In contrast, scholars in the fields of sociology and history and philosophy of science tend to cite a much lower percentage of articles: 40 % or lower. In other words: book citations are strongly linked with the humanities and social sciences.

Several researchers have investigated book citations. Tang (2008) analyses citations of 750 randomly selected monographs in the humanities and the sciences. Within each discipline, he finds differences in the number of uncited books, the time span in which half of the citations are occurring, and the recency of citations. In general, the fields of science tend to have lower numbers of uncited books and more recent citations compared to books in the humanities. However, the citation culture within each scholarly field is quite different. Nederhof (2011) deems the results of the impact investigations more useful, when a ‘citation window’ of at least six to eight years is used. According to Nederhof, this better reflects the world-wide reception of the publications. Another factor—not explicitly mentioned by Nederhof—is the fact that writing a book takes considerable more time than writing an article. This might have consequences for the citations in scholarly fields where monographs are the dominant publication form. Using a longer period to accumulate citations in the field of humanities is a solution also proposed by Linmans (2009). By doing so, Linmans is able to assess humanities publications. Furthermore, he expects Google Scholar to be a very useful source of book citations.

The use of Google Scholar as source of citation data is described by Harzing and Van der Wal. By comparing the coverage in the area of management and international business by Google Scholar and Thomson ISI Web of Knowledge, they conclude that Google Scholar is more comprehensive—especially in the area of books and non US journals (Harzing and van der Wal 2008). Whether Google Scholar or Google Books fares better than Scopus citations is tested by Kousha et al. (2011). Based on a set of 1,000 books, these authors determine that the larger amount of citations by the Google products could be used for assessing the publications in book-oriented disciplines in the British humanities and social sciences. More recently, Prins et al. investigated the coverage of social sciences and humanities by Web of Science (WoS) and Google Scholar. They conclude that the coverage by Google Scholar is better for these scholarly fields, although the quality of the data is not as consistent as WoS (Prins et al. 2014). In this paper, citations are derived from Google Scholar.

The availability of citation data for monographs is currently not on the same level as articles: the Thomson Reuters’ Book Citation Index was first published in 2011, providing citation information relating to a selection of just 25,000 titles (Jump 2011). The paucity of citation data relating to books within the prominent citation databases has inspired several authors to explore alternative sources of citation information. For instance, Kousha and Thelwall use the Google Books index to identify citations from books. Their goal is to compare the number of citations in the Thomson Reuters/Institute for Scientific Information databases (ISI) to those in Google Books. It is interesting to note that the ratios strongly differ between scholarly fields (Kousha and Thelwall 2009). This is in line with the conclusions of Nederhof, discussed earlier in this paper. Recently, Thelwall and Sud have used the Thomson Reuters Book Citation Index (BKCI) to explore whether co-authorship of monographs leads to a higher citation impact. Contrary to the results found for articles, the authors conclude that co-operation does not generally lead to more citations (Thelwall and Sud 2014). Again we see that citation behaviour for monographs differs from journal articles.

### Altmetrics

In the document “altmetrics—a manifesto”, altmetrics are described as an additional dimension to complement citation data. As publications are made available on the web, usage can be measured immediately (Priem et al. 2011). The online activities considered within altmetrics frameworks are diverse: a non-comprehensive list includes blog posts; tweets; Scopus citations; CiteULike bookmarks; Mendeley references or Facebook posts. The question of whether altmetrics measure usage from the academic world or should be treated of an indication of interest from wider reading communities will be discussed in the next “What is the relation between citations and altmetrics?” section.

In the realm of monographs and other book-length publications, several researchers have been working on alternative ways to assess scholarly value. Perhaps not surprisingly, data from academic libraries is used. For instance, White *et al.* discuss ‘libcitations’, where the number of academic libraries holding a certain book is the unit of measure. The collection of a library is based on qualitative decisions; a monograph that is acquired by a large number of libraries has a larger impact compared to a monograph that only resides in a few libraries. The authors do not compare those metrics to citation data (White et al. 2009; Zuccala and White 2015). In contrast, Cabezas-Clavijo et al. (2013) use the number of library loans from two academic libraries as a proxy of scholarly impact. When the library-generated data is compared with the available citation data, again the same pattern emerges: at best a weak correlation between the ‘alternative’ metrics and citations. Quite a different approach is used by Zuccala et al. (2014), who use machine-learning techniques to automatically classify the conclusions of book reviews in the field of history. However, the reported results derive from a pilot experiment, and no correlation to citations is described.

The question remains which altmetrics outlet to use to assess monographs. Here we face an additional complication: most altmetrics tools use an online unique identifier attached to a publication. In the case of journal articles, this will most likely be the Digital Object Identifier (DOI). Books are usually identified by an ISBN, but the use of ISBNs as digital identifier is not as widely spread as DOIs. This is especially true for the books in our data set. Another aspect to consider is the preferred outlet: are mentions of books evenly spread among all outlets? If that is not the case, which outlet or outlets are to be measured? Hammarfelt (2014) has compared the coverage in several online sources of 310 English language articles and 54 books—also written in English—in the field of humanities and social sciences. He concludes that for books, Twitter delivers the most results. In order to identify books, the title—or a significant part of the title—has been used.

### What is the relation between citations and altmetrics?

The relation between citations and altmetrics is currently under investigation. If these measurements are strongly correlated, they might measure something similar. However, if there is no strong connection, can altmetrics be considered to be an indication of a new aspect of impact? The literature discussed in this section is focused on journal articles; the connection between citation, altmetrics and books is poorly researched and there is little existing literature on the topic.

Several large-scale studies on correlations between citations and altmetrics have been performed. Using a set of over 24,000 open access articles published in the Public Library of Science, Priem et al. (2012) find a large uptake in at least one source of altmetrics activity. Yet, the correlation between citations and altmetrics is not very strong. Costas et al. (2014) arrive at a different conclusion regarding altmetrics activity: between 15 and 20 % of the articles in their set—based on more than 718,000 publications covered in the Web of Science—are mentioned via an altmetrics outlet, compared to almost 80 % in the case of Priem et al. (2012), who examined open access articles. Again, they do not find a strong connection between altmetrics and citations.

After a meta-analysis of seven studies, Bornmann (2014) concludes that different types of online outlets vary in the amount of correlation with citation counts. The bookmark counts of online reference managers Mendeley and CiteULike are the most connected to citations. In contrast, Twitter citations seem to measure something different from traditional citations: the correlation with traditional citations for the number of tweets is negligible. This is also described by Haustein et al. (2014), who conclude that Mendeley is predominantly used by the academic community, while Twitter is used by a general audience.

The report by Wouters et al. (2015) provides an overview of the current literature on the role of citations and altmetrics in research assessment. The report describes citations and altmetrics as complementary measures which should considered within the context of the publication. In a recent article by Thelwall (2016) the correlation between citations and altmetrics is also something to be considered within a certain context. Interpreting the correlation strength is quite complicated, as factors such as the average and the variability of the number of citations the documents received tend to play an important—but not always straightforward—role.

### Twitter as research tool

Using the number of tweets as an indicator of impact has several advantages when we look at the research at hand. Twitter is a widely used platform, which has been available since 2006. Due to its global usage and the extended period that it has been available for, we might expect more ‘success’ in identifying tweets about the books in our data set. The books in the data set analysed during this experiment were published between 1995 and 2008. The relatively long period between the publication of the books studied and the analysis carried out for this paper conforms to the longer ‘citation window’ discussed by Nederhof and Linmans. It may also allow for the accumulation of more tweets, which seems to be the case here. Moreover, Hammarfelt (2014) describes Twitter as the platform containing the most mentions of books, compared to other sources of altmetrics data. In the paper by Hammarfelt, the highest number of tweets for one book was 19. In our data set, 48 of the 400 books were mentioned in 25 tweets or more.

The results for this paper were derived using a search tool. While Twitter.com has its own search engine, a sample test performed in October 2014^2^ indicated that Topsy.com was more successful in identifying tweets about the books in the data set. This search engine had indexed all publically available tweets, making it a serious alternative to the Twitter.com search engine (Sterling 2013). Therefore, Topsy.com was used to identify relevant tweets for the purposes of this study.

### The influence of language

Little research is available on the influence of language on monograph citations. Abrizah and Thelwall (2014) have investigated—among other influences—the role of language in the number of citations Malaysian monographs received. While 71 % of the books analysed were published in Malay and the rest in English, the English language books were significantly more likely to be cited. Again, the authors have found differences between the citations in the different scholarly fields. Other researchers investigated the role of language on the citation rate of articles, by comparing the ‘native’ language to English (Aleixandre-Benavent et al. 2007; Guerrero-Bote and Moya-Anegón 2012; Winkmann et al. 2002). The common factor here is the bias of citation databases towards English, which disadvantages articles in other languages.

The relationship between language and Twitter usage has also been investigated. The paper by Hong et al. (2011) reveals the large proportion of English language tweets in the examined data set of over 62 million tweets. The number of tweets in English consist of 51 % of the total. This may also affect our outcomes, and we might expect more tweets for books in English, compared to the books in Dutch.

### The influence of subject

Nederhof (2011) describes citation impact measurements in modern language and linguistics research. Although these fields are closely connected, there are significant differences in publication and citation behaviour within each field. Whether the differences in citation patterns is also reflected in the number of tweets relating to books in different subject fields is not clear.

Holmberg and Thelwall (2014) examined a related question, by looking at disciplinary differences in how researchers use Twitter. This research was centred on all the tweets by scientists in ten disciplines. In contrast, this paper only examines tweets that mention the books in our data set. Holmberg and Thelwall conclude that differences in Twitter usage exist between scientific fields: those working on biochemistry, astrophysics, cheminformatics and digital humanities use it for scholarly communication. Others, who specialise in economics, sociology and history of science, are not deploying the microblogging site for their work. No information about the affiliation of the Twitter users in our data set is available, which makes it difficult to replicate this type of research.

## Research setup and the data set

The Introduction discussed whether publishing in open access has a significant effect on the scholarly impact of monographs, using citations and tweets. However, based on the literature review, we might expect additional influences by the scholarly field and language. Language is an important factor, as half of the collection analysed in this experiment is in Dutch, while the other half consists mainly of English language books. The study attempts to answer the research question, while taking into account these influences. Furthermore, we might expect a loose correlation between the number of citations and altmetrics. This is another aspect to be examined in this paper.

The data set consists of 400 books, all published by Amsterdam University Press (AUP), in the period 1995 to 2008. In the original experiment the books were divided into 4 sets of 100 titles (Snijder 2010). Three sets were immediately made available in open access; the fourth set was used as control and lacked full online availability. The books in the experimental data set were made available without embargo. Since the end of the experiment, the publisher has changed the availability of several books. The changes in availability since 2009 explain the percentages of OA in our data set: instead of 75, 68 % of the books are now freely available.

In the data set, 22 different subjects can be identified; in this data set we will treat the subject of the books as a proxy for scholarly field. The subjects are not evenly spread over the books: while 25 % of the titles discuss public administration and political science, the combination of the six topics education, economics, mathematics, theatre, information technology and religion accounts for just 6 % of the books.

In order to create groups of comparable size, the books were placed in two subject-based groups. Books on the subjects Archaeology, Art—History, Culture, Dutch Language, Education, History, Japan, Law, Literature, Motion Pictures, Music, Philosophy, Religion, and Theatre were included in the “Humanities” group. Books on Economics, Information Technology, Mathematics, Medicine, Psychology, Public Administration and Political Science, Science, and Sociology were placed in the “Other scholarly field” group (Table 1).

**Table 1:** Books in data set broken down for availability and subject

Accessibility	*N*	Percentage	Scholarly field	*N*	Percentage
Open access	271	68	Humanities	138	35
			Other scholarly field	133	33
Non OA	129	32	Humanities	82	21
			Other scholarly field	47	12

Compared to the number of subjects, the number of languages is quite small. More than half of the data set—212 books—comprises books published in Dutch; 178 books are published in English, while the remaining group of ten books are in either German or dual-language English-Dutch books. For the purposes of this study’s analysis, the books are divided in English-language titles and titles in other languages. The background section discussed the role of English; given the fact that only ten of the remaining books were not written in Dutch, they were not placed in a separate group (Table 2).

**Table 2:** Books in data set broken down for availability and language

Accessibility	*N*	Percentage	Language	*N*	Percentage
Open access	271	68	English	129	32
			Other languages	142	36
Non OA	129	32	English	49	12
			Other languages	80	20

Table 3 lists the combined data: the books divided into 8 groups.

**Table 3:** Books in data set broken down for availability, subject and language

Accessibility	*N*	Percentage	Subject and language	*N*	Percentage
Open access	271	68	Humanities—English	66	17
			Humanities—Other languages	72	18
			Other scholarly field—English	63	16
			Other scholarly field—Other languages	70	18
Non OA	129	32	Humanities—English	22	6
			Humanities—Other languages	60	15
			Other scholarly field—English	27	7
			Other scholarly field—Other languages	20	5

For complete details, please see the data set, available at http://dx.doi.org/10.17026/dans-x6m-67b2.

As mentioned in “Citations and books” section, the source of citations chosen for the purposes of this study is the Google Scholar website. In 2009, the citations were measured during the month August; in 2014 the citations were assessed in October. In the results section of this paper, the differences in citations will be discussed in more detail.

Most altmetrics tools use online identifiers—such as DOIs—to identify journal articles. Identifying publications turns out to be more problematic for monographs, which are more commonly associated with an ISBN. In contrast to DOIs, ISBNs are not widely used as an online identifier and searching for tweets using ISBNs did not prove to be successful. In contrast, searching for tweets using book titles delivered more results. Furthermore, personal communication with the founder of Altmetric.com has confirmed that—at the moment of writing—no online identifier for monographs can be used.^3^ In other words, a stable online identification was not available. However, searching for tweets using titles has disadvantages, particularly in relation to books that have been published in several editions. As far as could be established, the books in the data sets have not been published in several editions.

Apart from availability in open access, language and scholarly field have also been identified as having a possible impact on the number of tweets relating to any given title. According to the paper by Hong et al. (2011) half the tweets of their set containing 62 million tweets are sent in English. This may affect the number of tweets about books written in English as well. About half the books in our data set were written in Dutch. We can assume that these books will be read more by people in Dutch-speaking countries, while books written in English may attract a more global audience. Secondly, we have seen that within each scholarly field, the citation patterns are different. Does a comparable divide also exist in the use of social media? Are some subjects more prone to attract tweets than others? In our investigation, we will take both language and subject into consideration, combined with open access.

### Obtaining citations using Google Scholar

The citations were obtained using the same method as in the original experiment (Snijder 2010). For each of the monographs a URL pointing to a search at Google Scholar was constructed. The URL was based on the main title, placed between quotes. If needed, parts of the subtitle or the name of the author were added to ensure a best possible match. For instance, to find the book *Why Are Artists Poor?: The Exceptional Economy of the Arts*, by Hans Abbing, the following URL was used: http://scholar.google.com/scholar?hl=en&lr=&q=%22Why+Are+Artists+Poor?%22+The+Exceptional+Economy. This automatically opens the English language interface of the Google Scholar website, using the following search query: *“Why Are Artists Poor?” The Exceptional Economy*. Using quotes forces the website to search on the exact phrase; in this case, part of the subtitle was added to narrow down the results. The resulting number of citations was recorded.

The search was done manually, over several days. Restrictions on the Google Scholar website limit the number of searches that can be carried out within a short time. As was the case with searching for tweets, using the book title instead of the ISBN yielded the best results. The data set for this paper contains the search queries used on both Google Scholar and the Topsy.com website.

Each result was examined critically, and when multiple instances of a title—each containing their ‘own’ number of citations—were found, only the result with the highest number of citations was used. In the example below, the number of recorded citations was 25, not 29 (25 + 2 + 2). This method was also used in the experiment carried out in 2009.

- [BOOK] Reformation of Islamic thought: a critical historical analysis
  NHA Zayd—2006—books.google.com
  Cited by 25
- [CITATION] Reformation of Islamic Thought. A Critical Historical Analysis, wrr-Verkenning nr. 10
  N Abu Zayd—2006—Amsterdam: Amsterdam University…
  Cited by 2
- [CITATION] Nasr, (2006), Reformation of Islamic Thought: A Critical Historical Analysis, WRR/Den Haag
  A Zeyd—Amsterdam University Press,…
  Cited by 2

### Finding tweets using Topsy.com

The method used to find tweets resembles the routine for obtaining citations: again, the book titles were used in a manual process. In order to narrow down the results, quotes were used. For instance, the search for “The Rise of the Cult of Rembrandt” resulted in this URL: http://topsy.com/s?q=%22The%20Rise%20of%20the%20Cult%20of%20Rembrandt%22. Each query was set up in this way. If the search was not successful, the quotes were removed in an attempt to widen the search. All the resulting tweets were examined, and tweets on other subjects than the book in question were not counted. As mentioned before, neither ISBN nor another online identifier have been used. If readers tweeted a link to a book without using the title, this was not recorded.

## The results

As discussed earlier, this paper engages with the following research question: does open access have a positive influence on the number of citations and tweets a monograph receives, taking into account the influence of scholarly field and language? Additionally, the correlation between the number of citations and altmetrics activity has previously been investigated in relation to journal articles. Here, the correlation between monograph citations and tweets is investigated. Are they connected, or do these figures describe different aspects of impact?

**Fig. 1:**
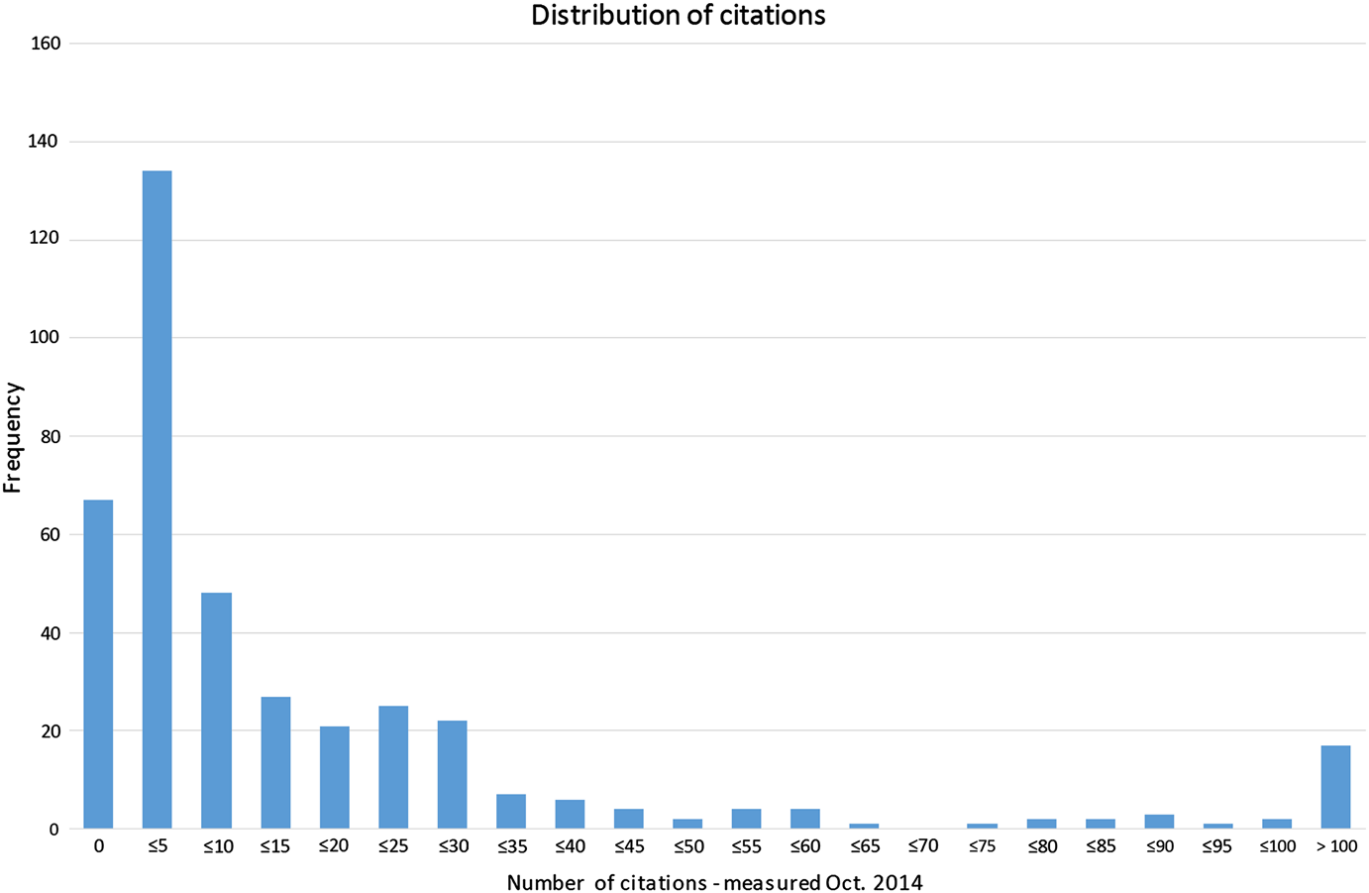
Frequency of citations, measured October 2014

**Fig. 2:**
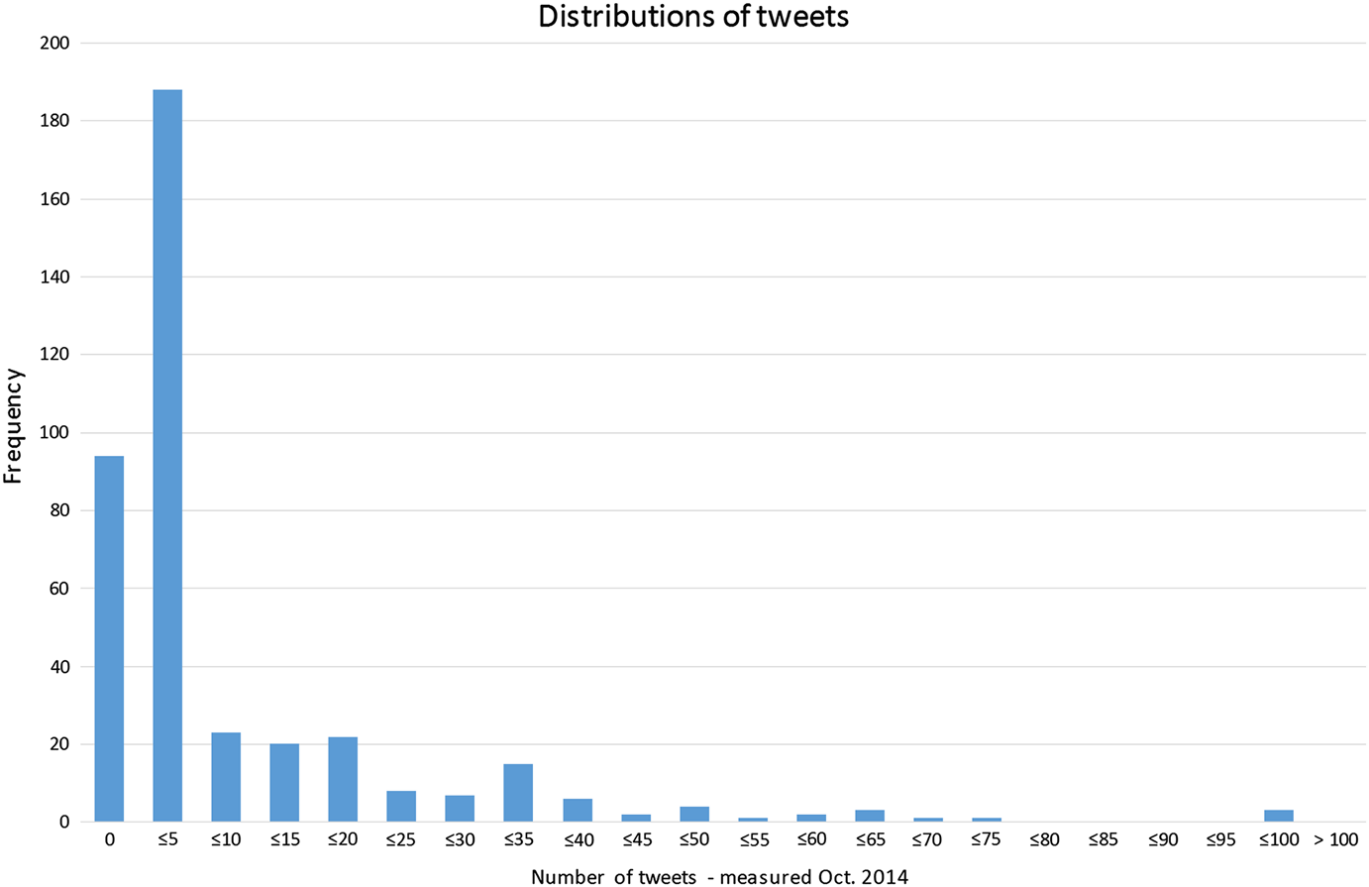
Frequency of tweets, measured October 2014

To get a sense of the way that citations and tweets are distributed across our data set, frequency has been plotted in two charts below. In both charts it is evident that distributions are skewed: most books received between one and five citations or tweets (Figs. 1, 2).

The books in our data set were published between 1995 and 2008. The literature cited in section 2 suggested that a ‘citation window’ of 6–8 years is preferable when assessing monographs. The effect of a longer period on the number of citations is clear in our data set. In 2009—the period of the original experiment—the average number of citations was 9.0. In 2014, the average has ascended to 39.0; more than four times as many. Perhaps more telling is the fact that in 2009, 183 of the 400 books received no citations. In comparison, the number of titles without citations has shrunk in 2014 to 67 books, meaning that 83 % of the books in the data set had been cited at least once.

The paper by Thelwall et al. (2013) predicts the opposite effect for altmetrics: due to a rapidly increasing uptake, newer publications will be mentioned more than older publications. However, Twitter was founded in 2006—more than a decade after some of the books in the sample examined for this project were published. Twitter analysis for this project was carried out in 2014. Although Topsy did capture historical tweets, the gap between the publication of many of the books in the sample and the advent of Twitter may partly explain the relatively low percentage of books with at least one mention on Twitter: 77 %.

The literature discussed earlier in this paper not only predicts more citations from a longer citation window, but it also describes differences in citation culture. Different citation patterns for different disciplinary areas are clearly visible in Table 4. The differences are also visible when books are categorised according to publication language in Table 5. The total number of citations counted in 2014 includes citations identified during the 2009 study; the column “tweets” lists the total number of tweets in which each monograph is mentioned.

**Table 4:** Books in data set broken down for subject: citations and tweets

Subject	*N*	Median (standard deviation)			Books with citations in 2014 (percentage)	Books with tweets (percentage)
		Citations 2009	Citations 2014	Tweets		
Humanities	220	0 (16.9)	4 (39.8)	2.5 (16.9)	157 (80)	172 (78)
Other scholarly fields	180	1 (103.8)	7.5 (211.7)	2 (12.8)	176 (98)	134 (74)
Total	400	1 (70.9)	5 (145.3)	2 (15.2)	333 (83)	306 (77)

**Table 5:** Books in data set broken down for subject: citations and tweets

Subject	*N*	Median (standard deviation)			Books with citations in 2014 (percentage)	Books with tweets (percentage)
		Citations 2009	Citations 2014	Tweets		
English	178	2 (104.8)	13 (213.5)	5 (15.6)	158 (89)	153 (86)
Other languages	222	0 (14.2)	3 (31.8)	1 (13.9)	175 (79)	153 (69)
Total	400	1 (70.9)	5 (145.3)	2 (15.2)	333 (83)	306 (77)

The literature on language and citations states that publications in English tend to receive more citations than texts in other languages. This may be in part because citation databases are more likely to index English language databases. In this paper, the citation data is not derived from a database such as Thomson ISI Web of Knowledge, but from Google Scholar. The sources indexed by Google Scholar are not known, and as such it is impossible to assess the extent to which Google Scholar citations are biased towards English language publications.

The influence of language—and the dominance of English—in relation to Twitter usage has also been discussed. In our data set, English language books are mentioned 13.2 times on average, while the average for books in other languages is far lower. Based on this, it seems reasonable to assume that the higher mean for English language books could partly be explained by the number of tweets in English.^4^

### Analysis of citations and tweets

To assess the relation between open access, language and subject, the data gathered has been analysed using a generalised linear model (GLM) analysis—in this case a negative binomial regression analysis. This type of statistical investigation allows for response variables that have error distribution models other than a normal distribution. We have seen that the distributions of both citations and tweets do not follow a neat ‘bell curve’, but are severely skewed. The GLM analysis is used to quantify the strength of the effect of the factors on the number of citations. Here, the factors are accessibility, language and scholarly field.

### Citations

The average number of citations for books published in OA was 35.7 (*SD* = 174.4); the mean number of citations for books not made available in OA was 13.4 (*SD* = 36.6). For the total set, the mean number of citations was 30.9 (*SD* = 157.44). Based on this, we might conclude that making books freely available has a large positive effect on the number of citations. If no further statistical analysis is deployed, the conclusion could be that the experiment has produced the expected result. This is also supported by the results of a negative binomial (maximum likelihood estimate) regression analysis. The estimated effect size Exp (B) with 95 % Confidence Interval (CI) is listed in Table 6. If only accessibility is taken into consideration, making books available in open access leads to 2.6 more citations (8 %)^5^ on average, compared to those published in closed access.

**Table 6:** Negative binomial regression: citations

	Exp (B)	95 % CI	
Accessibility (reference = non open access)			
Open access	2.588*	1.802	3.717
Intercept	14.884*	11.043	20.061

* Significant on 95 % level

However, when the effects of language and scholarly field are analysed, the results are more nuanced. Table 7 lists the results. When controlled for language and scholarly field, making a book freely available leads to 1.7 (5 %) more citations on average. However, the ‘citation advantage’ for books in English is 3.5 (11 %) and books in the humanities receive 0.5 citations on average (2 %), compared to books on other scholarly fields. The results still point to a slightly positive influence of open access on the number of citations, but the effects of language and scholarly field are also significant.

**Table 7:** Negative binomial regression: citations, language, scholarly field

	Exp (B)	95 % CI	
Accessibility (reference = non open access)			
Open access	1.657*	1.168	2.352
Language (reference = other languages)			
English	3.509*	2.529	4.869
Scholarly field (reference = other scholarly fields)			
Humanities	0.538*	0.391	0.740
Intercept	12.757*	8.920	18.243

* Significant on 95 % level

#### Tweets

The average number of tweets for books published in OA was 9.1 (*SD* = 15.4); the mean number of tweets for books not made available in OA was 7.6 (*SD* = 14.6). For the total set, the average number of tweets was 7.86 (*SD* = 16.044). Again, at a first glance we see an advantage for OA books and we might be tempted to conclude that publishing monographs in open access leads to a higher uptake by social media, in this case Twitter. Nevertheless, this conclusion is refuted by the results of a negative binomial (maximum likelihood estimate) regression: when only open access is considered, the results are not statistically significant (Table 8).

**Table 8:** Negative binomial regression: tweets

	Exp (B)	95 % CI	
Accessibility (reference = non open access)			
Open access	1.188	0.806	1.751
Intercept	6.977*	5.068	9.605

* Significant on 95 % level

The results of Table 9 show that the effects of language and scholarly field are statistically significant, in contrast to accessibility. Books in English receive 2.5 (31 %) more tweets and books in the humanities get 1.8 more tweets (22 %) on average.

**Table 9:** Negative binomial regression: tweets, language, scholarly field

	Exp (B)	95 % CI	
Accessibility (reference = non open access)			
Open access	1.211	0.827	1.772
Language (reference = other languages)			
English	2.454*	1.697	3.549
Scholarly field (reference = other scholarly fields)			
Humanities	1.779*	1.224	2.585
Intercept	3.032*	1.929	4.766

* Significant on 95 % level

Based on the literature, we might expect that both subject and language are significant factors, whether or not the books have been made available in open access; if different scholarly fields have different citation cultures, this should affect the outcomes. The results point to the same effect on tweets. Yet, we could argue that analysing citations from different scholarly fields is comparing apples and oranges: within each discipline, the average number of citations is different. This may have impacted the results of the analysis. In order to compensate for discipline variance, it is necessary to compare the number of citations and tweets within a group of books with the same subject. The results of this analysis are described in the next section.

### Statistical analysis within subject

The books in the data set are not evenly distributed across subjects. While 98 books discuss the subject “Public Administration and Political Science”, there are also groups of just three or four books on subjects such as “Economics”, “Mathematics”, “Theatre” or “Religion”.

If the mean number of citations and tweets are plotted on a chart, the differences become visible in a literal sense: the mean number of citations differs between scholarly fields, and a high number of mean citations is not matched by a high number of tweets. The chart also lists the number of books per subject. Whether the results of analysing subject-based groups containing as little as four or three books have any statistical significance is highly doubtful.

For this reason, only the five largest subject-based groups are analysed, again using the negative binomial procedure. The following subjects were examined using this approach: “Public Administration and Political Science”, “Literature”, “History”, “Sociology” and “Motion Pictures”. The total number of books in the five largest subject-based groups is quite large: 238 titles. Of those titles, 172 were published in open access, and 66 were not made openly available (Table 10).

**Table 10:** The five largest subject-based groups: number of titles, citations and tweets

Subject	Open access books			Non open access books		
	*N*	Median citations (SD)	Median tweets (SD)	*N*	Median citations (SD)	Median tweets (SD)
Public Administration and Political Science	82	10.5 (40.6)	2 (8.6)	16	6 (95.5)	2 (24.6)
Literature	19	2 (7.0)	1 (28.4)	20	2 (13.0)	0.5 (27.3)
History	22	4.5 (58.4)	2.5 (17.8)	15	4 (10.0)	1 (5.8)
Sociology	22	18 (31.7)	0.5 (8.5)	11	7 (18.5)	2 (11.0)
Motion Pictures	27	24 (44.5)	15 (13.8)	4	21 (2.9)	14.5 (10.3)

Measured October 2014

The results of the citation analysis based on the five subjects are mixed. In the case of the books on “Literature” and “Sociology”, neither accessibility nor language are statistically significant. Language is a significant factor for “Public Administration and Political Science” and “Motion Pictures”. Only for “History”, open access was a statistically relevant factor.

The results of the tweet analysis based on the five subjects follow a different pattern compared to citations. Here, neither accessibility nor language were statistically significant for “Public Administration and Political Science”, “Literature” and “Motion Pictures”. Language is a significant factor for “Sociology”, and—as is the case with citations—accessibility is significant for “History”.

Taking these results into account, the conclusion must be that open access does not affect significantly the number of tweets relating to a specific title. However, the influence of language is also limited. Again, the data set is available at http://dx.doi.org/10.17026/dans-x6m-67b2.

### Correlating citations and tweets

The background section of this paper describes work by Priem et al. (2012) and by Costas et al. (2014), in which correlation analysis was used to test for a connection between the number of citations associated with journal articles and altmetrics activity. Of course, correlation is not causation and a connection between citations and altmetrics does not imply such a simple relation. However, a strong correlation may suggest an underlying cause. Both papers reported a weak but positive correlation between citations and altmetrics activity. In other words, when citations are higher, there is a small chance that altmetrics activity will also be higher.

**Fig. 3:**
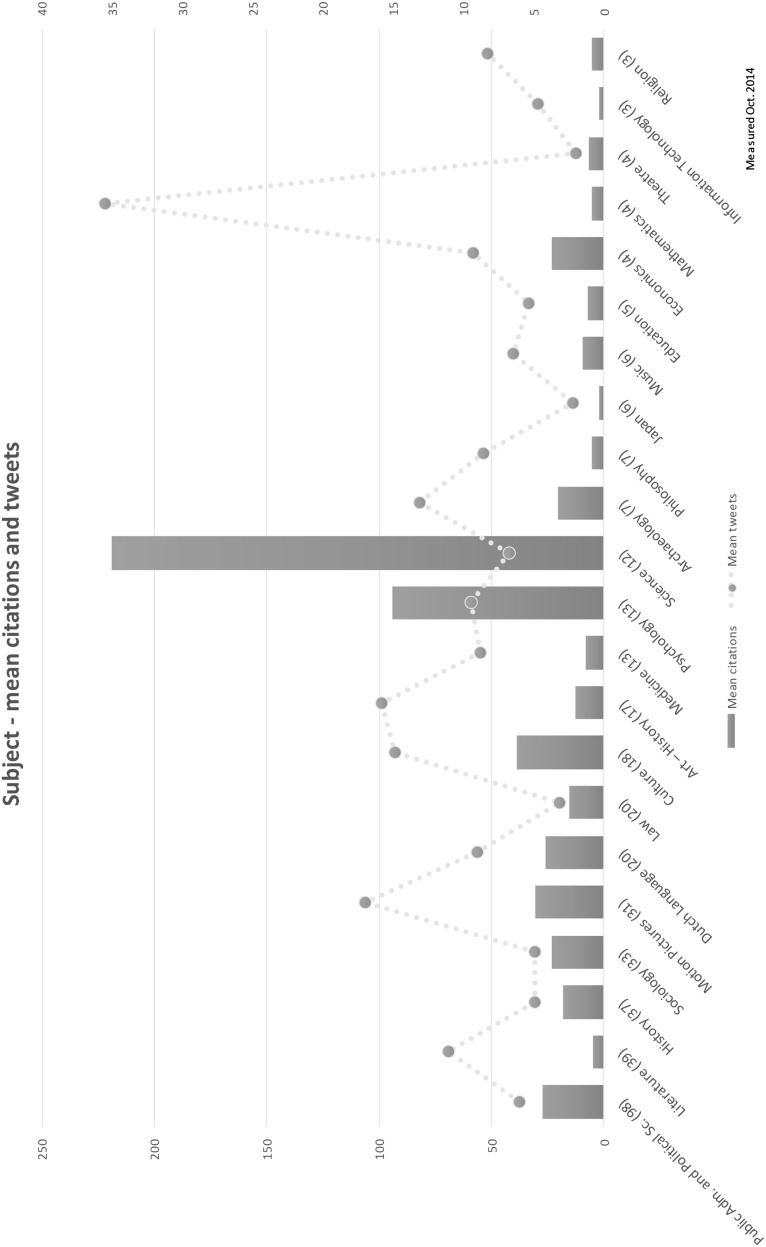
Mean citations and tweets—per subject

Haustein et al. (2014) link Twitter to a general audience. The altmetrics data in this paper consists of Twitter data, and we might expect only a weak correlation between citations and tweets. In other words: the measured usage of scholarly output—for which the number of citations is used as a proxy—might differ considerably from the interest expressed by the general public—for which the number of tweets is used as a proxy. Lastly, Fig. 3 shows the differences in mean citations and tweets for books with the same subject. This also is an indication of a weak correlation.

A Spearman’s correlation has been computed to determine the relationship between the number of citations and tweets in the data set. There was a moderate, positive correlation between citations and tweets (*r*_*s*_ = .299, n = 400, *p* < .001). While keeping in mind the uncertainties described by Thelwall (2016), this result is consistent with the idea that there is not much overlap between academic usage and the interest of a general audience.

## Conclusions

The 2009 monograph experiment was set up to measure the influence of open access using several indicators. During the 9 months the experiment ran, it became clear that discovering and consulting the books online benefits strongly from open access. According to the literature on journal article citations and open licenses, a positive influence on monograph citations should have been expected. However, the effect did not occur in 2009. Five years later, the freely accessible books had been cited more on average compared to the control group; a result that confirms the hypothesis that open access has an effect on citations. Yet, when statistical analyses are deployed, the results are more nuanced: when differences in language and subject were controlled for, a small positive effect of OA publishing on citation scores remained.

One of the propositions of making scholarly documents freely available is that it widens access, including for academics who would otherwise not be able to read them. From this follows the assumption that more academic readers will eventually cite the document in their own work. The 2009 experiment demonstrated that online usage benefits from open access, but this usage did not result in more citations. Measuring citations 5 years later allowed for the longer time period associated with writing books, which are still a major publication form in the humanities and social sciences. The number of citations measured in 2014 revealed a slight citation advantage for open access books.

A possible explanation can be found in the results of the OAPEN-UK survey of British scholars. Most of the respondents declared that they had little trouble in accessing relevant books, either by borrowing or buying them. Here at least is no indication of diminished access to monographs. As the most likely readers of Dutch language monographs, scholars in the Netherlands and Belgium might work under comparable conditions with relative easy access to academic libraries or funds to purchase books. If that is the case, the significance of free access to online books becomes smaller, although open access might still enhance access.

This study found a similar relationship between open access, subject and language on altmetrics activity associated with books. In the case of OA monographs, making them freely available had a clear positive effect on usage: the free books were used more when compared to a control group of books that are not available in open access. This higher usage has translated into a higher uptake in social media, although the effects of subject and language again played an important role. However, the higher uptake for freely accessible books is not statistically significant.

The results identified very little overlap between Twitter usage and citation behaviour; it seems reasonable to hypothesise that the factors affecting citations of books do not play a significant role in tweets about books. Therefore, the probable reason that open access is a significant influence on book citations does not necessarily apply to Twitter mentions. Nonetheless, it is possible to conclude that making books freely available has some positive impact on the number of tweets. Lowering the access barrier does indeed lead to more attention, in line with the effects for discoverability and online consultation found in the 2009 experiment.

The results also point to the fact that barriers to access are not the only reason for lack of attention. Within the formalized realm of scholarly discourse, the mean number of citations tends to be closely connected to the scholarly discipline. The mean number of tweets per discipline does not follow the same pattern, but there are certainly subjects which are more popular than others. Books on literature, motion pictures or history of art receive a higher number of tweets on average, compared to subjects like history, sociology or law. In other words, the impact of subject should be filtered out, before the effect of open access can be measured.

Apart from subject, language is another factor. This plays a large role in determining the number of both citations and tweets. Publishing books in languages other than English does not only affect usage by scholars, but also the uptake on Twitter. The latter is easily explained by the current preference for English as lingua franca, but also by the fact that scholars are less likely to give attention to other languages. Whether this result is specific to this data set only—because it includes a large portion of Dutch language books—or whether the same result would be found in collections containing a different mix of languages remains to be seen.

## Further investigation: beyond the OA citation advantage?

This paper attempts to shed light on the effects of open access on books, rather than on journal articles. The paper has identified that the effect of OA is not as profound for books as it appears to be for journal articles, and that further examination of differences between books and journal articles is warranted. An area of particular interest for future research is whether the slower publication cycle associated with books changes the effect of open access, or whether other factors such as disciplinary culture are responsible for apparent differences.

Another way of looking at the results might be that the OA citation advantage exists, both for articles and books. This has been demonstrated in the case of journal articles again and again. However, more research on the effects of OA on monographs would be welcome, as the amount of published research in this area is small. Still, more interesting questions can be asked.

For instance, if open access helps to disseminate scholarly publications beyond the more affluent academic organisations, will citations and altmetrics reflect this? In other words: will freely available publications be cited more often by scholars working in less privileged circumstances? Or does open access only favour those who would have access anyway? This question could also be investigated through the lens of altmetrics, with a view to establishing whether or not the altmetrics indicators measured are associated with a wider, more global audience.

Earlier in the paper the connection between citation and altmetrics behaviour was discussed. While directly interrogating the reasons for citations or online activities is a complex challenge, this is also an important direction for future research. Understanding whether there are differences between the ways in which research communities perceive OA documents when compared to closed equivalents may shed light on differences in altmetrics and citation profiles.

Lastly, if the importance of bibliometric analysis as a proxy for research quality is growing, it is vital to understand if there are significant dissimilarities between articles and monographs. Identifying specific differences between journal articles and books and the factors that underlie these differences will enable a comparison of scholarly impact of monographs and articles based on sound principles.

## Limitations

For the purposes of this study tweets referring to book titles were identified through the altmetrics search engine—Topsy.com. The limitations of the Topsy search engine are not known. Furthermore, searching for a book’s title may be an imperfect way to find all mentions, due to a lack of online identifiers for monographs.

The method used to collect tweets was geared towards quantitative results: apart from removing tweets on subjects other than the book in question, no attempt was made to analyse the content of individual tweets. Whether authors actively participated in the promotion of their books via social media is not known. However, the author of this paper was employed at Amsterdam University Press from 2007 to 2014. During that period, no formal policy existed for promoting publications by authors using social media.

Within the analysis, factors other than language and subject were not been corrected for. For instance, the role of document length or publisher’s prestige were not accounted for.

## Footnotes

1 See for instance Sect. 1.2.3. *Normalisation of citation impact indicators* of Wouters et al. (2015).

2 The Topsy.com service has been discontinued in December 2015.

3 Euan Adie, personal communication, 10 February 2014.

4 The dataset used does not record the language of the tweets.

5 Throughout Sect. 4, the average number of citations/tweets of that data set is used as reference.
